# Snakebite Causing Facial and Lingual Tremors: A Case Report

**DOI:** 10.7759/cureus.27798

**Published:** 2022-08-08

**Authors:** Nidhi Kaeley, Hari Prasad, Ashutosh Singhal, Soumya Subhra Datta, Santosh S Galagali

**Affiliations:** 1 Emergency Medicine, All India Institute of Medical Sciences, Rishikesh, Rishikesh, IND

**Keywords:** tremors, snakebite, mechanical ventilation, involuntary movements, calcium gluconate

## Abstract

Snakebite is a significant public health problem causing around 2.7 envenomations and 138,000 deaths globally. History may sometimes be unclear or misleading, which can cause a delay in diagnosis. Neuroparalytic, hemotoxic, and myotoxic are the common snake bite manifestations. Neuroparalytic snake bites rarely cause involuntary movements. Here we report a case of a 26-year-old female patient who sustained a snake bite and developed tremors in the face and tongue. She improved with mechanical ventilation, anti-snake venom, atropine-neostigmine, and calcium gluconate. She was discharged after seven days of hospital stay and now maintaining regular follow-up in the outpatient clinic.

## Introduction

World Health Organization (WHO) reinstated snakebite envenoming to its list of categories as a neglected tropical disease (NTDs) in 2017 as there was a higher incidence and severity of snakebite worldwide with uneven accessibility to antisnake venom, its primary management [1]. The cobra and krait venoms are neurotoxic and cardiotoxic. Viper venom is vasculotoxic and has severe necrotizing local effects. The presentations can be local or systemic [2]. There are only a few case reports on involuntary movements following snakebite; Here, we describe a 26-year-old female who developed tremors following snakebite which later responded well to medical treatment.

## Case presentation

A 26-year-old female with no known addictions or comorbidities presented to the emergency department of a tertiary care center in India at around 6:30 am with a history of unconsciousness. The patient was found unconscious in her room at around 5:45 am by her relatives with multiple episodes of nonbilious nonblood stained vomiting and nonblood stained, nonfoul smelling loose stools. The patient was last seen well the previous day around 7 pm by her husband. There were no associated seizures, bleeding from any body site, or external trauma. She was initially taken to a local hospital where organophosphorus poisoning was suspected, and the patient was given atropine and pralidoxime and was referred here.

On clinical examination, the patient was unconscious. Her vital signs were blood pressure of 114/70 mm Hg, heart rate of 84 beats/minute, oxygen saturation of 98% on room air, respiratory rate of 18 breaths/minute, and random blood sugar of 180mg/dL. Her Glasgow Coma Scale (GCS) score was E1V1M4. The patient's bilateral pupils were reactive and of normal size. In central nervous system examination, power in all four limbs was 5/5. The tone was normal, and the bilateral plantar was flexor. There was no cranial nerve involvement or cerebellar signs. Other system examinations were within normal limits.

Blood investigations revealed neutrophilic leucocytosis, prolonged prothrombin time, and international normalized ratio. Rest blood investigations were within normal limits, as shown in Table 1. Noncontrast head CT was normal. Due to atypical presentation and unmatched clinical signs, an unresponsive patient was made a broader differential diagnosis. Snakebite was also considered as one differential, and a whole blood clotting time was done that came to be more than 20 minutes.

**Table 1:** Blood test results ALT, Alanine aminotransferase; AST, Aspartate aminotransferase; ALP, Alkaline phosphatase; INR, International Normalized Ratio

Parameters	Values	Reference range
Hemoglobin	11.9 g/dL	12–15 g/dL
Red blood cell count	5.18 × 10^6^/mcL	3.8–5.2 × 10^6^/mcL
White blood cell count	21.41 × 10^3^/mcL	4–11 × 10^3^/mcL
Platelets	356 × 10^3^/mcL	150–400 × 10^3^/mcL
Total bilirubin	0.82 mg/dL	0.3–1.2 mg/dL
Direct bilirubin	0.14 mg/dL	0–0.2 mg/dL
Alanine aminotransferase (ALT)	34 U/L	0–35 U/L
Aspartate aminotransferase (AST)	29 U/L	0–35 U/L
Alkaline phosphatase (ALP)	158 U/L	30–120 U/L
Serum albumin	4.0 g/dL	3.5–5.2 g/dL
Urea	21.2 mg/dL	17–43 mg/dL
Creatinine	0.86 mg/dL	0.55–1.02 mg/dL
Sodium	136 mEq/L	136–146 mEq/L
Potassium	4.8 mEq/L	3.5–5.1 mEq/L
Calcium	8.46 mg/dL	8.8–10.6 mg/dL
Prothrombin time	23.2 sec	13.6 sec
International Normalized Ratio (INR)	1.41	0.90–1.20

The patient was intubated because of airway protection using etomidate and rocuronium. The patient was given ten vials of anti-snake venom around 8 am because of whole blood clotting time was more than 20 minutes. The patient's sensorium improved by 4 pm that day, but the patient had ptosis on examination. So patient was given five doses of atropine (0.6mg), and neostigmine (1.5mg) intravenously repeated every 30 minutes and ten more vials of anti-snake venom. The patient was extubated the next day as ptosis improved. Post extubation patient started developing tremors of the face and tongue. Video 1 (https://vimeo.com/734789312) shows facial and lingual tremors in the patient.

**Video 1:** Facial and lingual tremors in the patient

So five more doses of atropine-neostigmine combination and ten more vials of anti-snake venom were repeated. However, tremors persisted, so 10ml 10% calcium gluconate was given stat every six hours intravenously. After three days of continuous calcium gluconate tremors were relieved. The patient was discharged on day seven of admission as there was taking orally, no ptosis or any breathing difficulty, a single breath count of 40 per min, and no bleeding manifestations or rhabdomyolysis. The patient is now maintaining regular follow-up in the out patients department.

## Discussion

Snake venom contains more than 20 toxins, including enzymes, non-enzyme peptides, and non-toxic proteins. Local skin features include fang marks, pain, local swelling, local necrosis, and secondary infection. Systemic features can be hemotoxic, myotoxic, or neurotoxic [2]. Hemotoxic manifestations include bleeding from the puncture site, epistaxis, ecchymosis, hematemesis, hemoptysis, subconjunctival hemorrhage, and retroperitoneal bleed. Myotoxic manifestations include myalgias, myopathy, rhabdomyolysis, generalized aching, stiffness, and tenderness of muscles. Neurotoxic manifestations include starting with ptosis and external ophthalmoplegia. Later the face, tongue, palate, jaws, vocal cords, muscles of deglutition, and neck muscles become paralyzed. Obstruction of the airway or paralysis of the diaphragm causes respiratory failure [3]. 

The neurotoxins can be either presynaptic or post-synaptic, which causes paralysis by interfering with the neuromuscular transmission, but they do not cross the blood-brain barrier. Beta-neurotoxins like phospholipase A2 complexes belong to a class of presynaptic neurotoxins that inhibit the release of acetylcholine from the presynaptic terminal and interfere with the formation of new acetylcholine vesicles. Examples include taipoxin, trimucrotoxin, textilotoxin, viperotoxin, paradoxyn, pseudocerastes, and crotoxin. Alpha neurotoxins include three-finger protein complexes that belong to a class of post-synaptic neurotoxins and have curare-like action, thereby causing reversible blockade of acetylcholine receptors. Complex blockage of neuromuscular transmission is caused by venoms containing alpha and beta neurotoxins [4].

Involuntary movements following snake bites are less commonly reported. Entirely reversible effects are the neurotoxic ones that wear off either acutely in contact with anticholinesterases or anti-snake venoms or spontaneously in one to seven days. A possible explanation for involuntary movements can be due to the interaction of snake venom with voltage-gated potassium or calcium channels in the axons of the nerve [5]. The exact magnitude of the involuntary movements in snakebites is unknown.

The first reported involuntary movement was facial and limb myokymia in timber rattlesnake envenomation by Brick et al. in 1987. The case report suggests that the biochemical mechanism of venom action increased peripheral nerve excitability as facial myokymia responded to anti-snake venom and limb myokymia by increasing serum ionized calcium [6].

Lo Vecchio et al., in 2005, reported two cases of myokymia from Arizona; the first case was of a 14-year-old boy who presented with complaints of generalized myokymia over the face and limbs after a rattlesnake bite. There was no evidence of significant coagulopathy. The involuntary movements resolved with 10 vials of antivenom. The second case was that of a 40-year-old female who presented with myokymia and perioral paresthesias after a rattlesnake bite. The myokymia resolved with calcium chloride (2g of 10%). These observations indicate the role of snake venom and calcium channel interaction [7].

Another case of diffuse myokymia involving thigh muscles was reported by Ramcharan et al. in 2016. The author concluded by saying myokymia predicts mechanical ventilator need [8]. A recent case report of myokymia by Garg et al. in a 23-year-old farmer improved with 0.6mg atropine and 1.5mg neostigmine in five doses and 20 vials of anti-snake venom [9].

Another involuntary movement reported was Lance-Adams syndrome in a 21-year-old male who developed progressive respiratory failure and mechanical ventilation following snakebite. The patient developed involuntary movements post-extubation [10].

Tremors involving the face, tongue, or other body parts have not been reported. The possible explanation of tremors will be the interaction between snake venom and myoneural junction as the involuntary movements improved with calcium, anti-snake venom, and atropine-neostigmine combination.

## Conclusions

The exact cause of involuntary movements like tremors and myokymia in snake bites remains unknown. So further studies are required on this topic. Involuntary movement could be an early predictor of the need for a mechanical ventilator. The treatments that can be tried are calcium, anti-snake venom, and atropine-neostigmine.
